# 

**Published:** 1846-03

**Authors:** 


					﻿TERMS, ONE DOLLAR A YEAR, IN ADVANCE.
THE
äoaojis
MEDICAL & SURGICAL
JOURNAL.
EDITED BY
JAMES V. Z. BLANEY, M. D.
Professor, &c.
CHICAGO, ILL.
PUBLISHED BY ELLIS & FERGUS,
BOOK AND JOB PRINTERS,
SALOON BUILDINGS, CLARKE STREET,
1846.
VOLII.
NO. 12.
				

